# Emerging highly pathogenic H5N1 influenza triggers fibrotic remodeling in human airway organoids

**DOI:** 10.1080/22221751.2025.2532684

**Published:** 2025-07-25

**Authors:** Hussin Rothan, Ahmed Mostafa, Mahmoud Bayoumi, Chengjin Ye, Ramya S. Barre, Anna Allué-Guardia, Aitor Nogales, Jordi B. Torrelles, Luis Martinez-Sobrido

**Affiliations:** a Host-pathogen interactions (HPI) and Disease Intervention and Prevention (DIP) Programs, Texas Biomedical Research Institute, San Antonio, TX, USA; b International Center for the Advancement of Research and Education (I•CARE), Texas Biomedical Research Institute, San Antonio, TX, USA; c Population Health Program, Tuberculosis Group, Texas Biomedical Research Institute, San Antonio, TX, USA; d Center for Animal Health Research, CISA-INIA-CSIC, Madrid, Spain

**Keywords:** Human airway organoids, influenza virus, prolonged infection, fibrogenesis, ROCK signalling pathway

## Abstract

The ongoing outbreak of highly pathogenic avian influenza (HPAI) H5N1 clade 2.3.4.4b has affected at least 989 dairy herds across 17 states in the United States (U.S.) and resulted in 70 confirmed human infections, underscoring the urgent need to understand the pathogenesis and therapeutic interventions of emerging H5N1 viruses. In this study, we modelled infection with a highly pathogenic recombinant human A/Texas/37/2024 H5N1 (rHPh-TX H5N1) strain using human airway organoids (HAO) to investigate viral replication, innate immune response, infection-induced fibrogenesis, and potential therapeutic interventions. rHPh-TX H5N1 replicated efficiently in HAO, eliciting a robust interferon (IFN) response and pro-inflammatory cytokine production. Prolonged infection led to the accumulation of fibroblast-like cells surrounding infected regions, marked by increased alpha-smooth muscle actin (α-SMA) expression and upregulation of transforming growth factor-beta (TGF-β), indicative of fibroblast activation and extracellular matrix (ECM) remodelling. Compared to organoids infected with the pandemic A/California/04/09 H1N1 (pH1N1) strain, rHPh-TX H5N1 induced significantly higher expression of fibrosis-associated markers, including fibronectin (FN), collagen 1A (COL1A), collagen 3A (COL3A), metalloproteinases 2 and 9 (MMP2, and MMP9). Notably, the inhibition of Rho-associated coiled-coil-forming protein kinases (ROCK) signalling reduced fibrogenesis, with ROCK1 inhibition being more effective than ROCK2 inhibition. These findings highlight the potential of targeting ROCK signalling to mitigate H5N1-induced lung fibrosis, informing therapeutic strategies for severe influenza infections.

## Introduction

Influenza A virus (IAV) pandemics have historically represented significant global health challenges, characterized by widespread morbidity, high mortality, and profound social disruption [1]. These pandemics arise when novel IAV strains emerge, typically through genetic reassortment between human and animal influenza viruses, causing new strain variants to evolve with limited pre-existing immunity in the human population [2–4]. Notable IAV pandemics include the 1918 “Spanish flu,” which caused an estimated 25–50 million deaths worldwide, the 1957 “Asian flu,” the 1968 “Hong Kong flu,” and, more recently, the 2009 “swine flu” caused by H1N1 [5–7]. In the spring of 2024, a multistate outbreak of a highly pathogenic H5N1 influenza virus strain, originating from both human and bovine sources, was reported across dairy cattle farms in the United States (U.S.). This emergent strain, referred to as highly pathogenic human and bovine Texas H5N1 (HPh-TX H5N1 and HPb-TX H5N1, respectively), led to several confirmed human infections [8], raising significant public health concerns. Experimental studies in non-human primates have demonstrated the virulence of bovine H5N1 clade 2.3.4.4b, including the development of severe disease, with widespread viral dissemination and extensive lower respiratory tract damage, mimicking the clinical progression seen in severe human cases [9]. These findings underscore the potential of this emergent virus to cause both zoonotic transmission and life-threatening respiratory illness.

Acute IAV infection is often characterized as a self-limiting respiratory illness, with symptoms such as fever, cough, and malaise resulting from the immune response to viral replication. However, severe cases may occur, particularly in individuals with compromised immunity, pre-existing health conditions, or infections involving highly pathogenic strains such as H5N1 and H7N9. Complications can arise in some cases, including viral pneumonia, acute respiratory distress syndrome (ARDS), and multi-organ failure [7,8]. Although prolonged IAV infection is rare, it is more common in immunocompromised patients, leading to persistent viral shedding, increasing vulnerability to secondary microbial infections, and impaired tissue repair. These factors contribute to long-term complications such as lung injury and fibrosis [10,11]. Therefore, mechanistic insights into the interplay between viral replication, immune responses, and tissue repair are essential for managing severe IAV infections and mitigating their long-term consequences.

One critical complication of severe IAV infection is lung fibrosis, a debilitating and often irreversible condition characterized by excessive extracellular matrix (ECM) deposition and impaired lung function [12]. While most individuals recover from IAV infection without lasting damage, infections with highly pathogenic influenza strains or severe disease can disrupt the epithelial tissue architecture, induce chronic inflammation, and activate profibrotic pathways, such as transforming growth factor-beta (TGF-β) signalling [13,14]. Furthermore, persistent epithelial injury, fibroblast activation, and immune cell recruitment further exacerbate fibrotic remodelling [15]. Therefore, investigating these pathological processes is crucial for identifying therapeutic targets and preventing long-term pulmonary dysfunction following IAV infections.

Human airway organoids (HAO) are being used to assess the spillover risk of highly pathogenic avian influenza H5N6 and H5N8 isolates [16]. This model has also emerged as a powerful system for investigating lung fibrosis mechanisms and exploring potential therapies [17]. HAO possesses the key features of lung architecture for studying the fibrotic changes after exposure to pro-fibrotic stimuli like TGF-β, ECM remodelling, and fibrogenic signalling pathways [18,19]. While current limitations include incomplete immune system integration and challenges with long-term maintenance, lung organoids represent a transformative tool for understanding lung fibrosis and accelerating therapeutic discovery. Therapeutic strategies for virus-induced lung fibrosis focus on mitigating tissue remodelling and preserving lung function. Antiviral treatments may indirectly reduce fibrosis by suppressing the inflammatory response initiated by viral replication. Drug treatment programmes for virus infection-related lung fibrosis are currently formulated based on the relevant guidelines for idiopathic pulmonary fibrosis (IPF), although there is no clear drug treatment programme recommendation.

In this context, Rho-associated coiled-coil-forming protein kinases (ROCK1 and ROCK2) play critical roles in many cellular responses to injury [20]. In this study, we utilized HAO to model infection with recombinant rHPh-TX H5N1 strain, investigating its effects on viral replication, innate immune responses, and the development of infection-associated lung fibrosis. The virus demonstrated efficient replication within the HAO, triggering a potent interferon-mediated antiviral response and an increase in the expression of pro-inflammatory cytokines. Prolonged infection with rHPh-TX H5N1 caused myofibroblast differentiation, marked by increased expression of alpha-smooth muscle actin (α-SMA). Additionally, rHPh-TX H5N1 infection was associated with the upregulation of TGF-β, which facilitated fibroblast activation and ECM remodelling evidenced by measuring the expression levels of fibronectin (FN), collagen 1A (COL1A), collagen 3A (COL3A), metalloproteinases 2 and 9 (MMP2, and MMP9). Notably, inhibition of the ROCK pathway attenuated α-SMA expression, reduced myofibroblast differentiation, and suppressed ECM protein expression, highlighting its potential as a therapeutic target to mitigate IAV-related lung injury and fibrosis. Altogether, rHPh-TX H5N1 infection induces a robust inflammatory and fibrotic response, driven by NF-κB and TGF-β signalling, which promotes airway remodelling and fibrosis. Targeting ROCK1 may offer a potential strategy to mitigate IAV-induced lung fibrosis.

## Methods

### Biosafety

All experiments with the pandemic A/California/04/09 H1N1 (pH1N1) and highly pathogenic human and bovine Texas H5N1 (HPh-TX H5N1 and HPb-TX H5N1, respectively) were conducted at the Texas Biomedical Research Institute under biosafety level 2 (BSL-2) or BSL-3, respectively, and were approved by the institutional biosafety committee (IBC).

### Cell lines

Commercial human lung adenocarcinoma epithelial A549 (ATCC CCL185, Manassas, USA) and Madin-Darby canine kidney, MDCK (ATCC CCL-34, Manassas, USA) cells were maintained in Dulbecco’s modified Eagle medium (DMEM) (Invitrogen, Waltham, USA) supplemented with 10% fetal bovine serum (FBS) and 1% PSG (penicillin, 100 U/mL; streptomycin 100 μg/mL; L-glutamine, 2 mM) at 37°C in a humidified 5% CO_2_ incubator.

### Viruses

rHPh-TX H5N1 and rHPb-TX H5N1 were generated by reverse genetics [21]. The rHPh-TX H5N1 non-structural 1 (NS1) C-terminus was fused either to the mCherry or Venus fluorescent proteins, to generate pH1N1-mCherry and rHPh-TX H5N1-Venus, respectively, as previously described [21]. Briefly, NS1 segments were synthesized *de novo* (Bio Basic, Amherst, USA) with the appropriate restriction sites for subcloning into the ambisense pDZ (pH1N1) and pHW2000 (rHPh-TX H5N1) plasmids. Modified NS1 segments contain the open reading frame (ORF) without stop codons or splice acceptor sites, followed by the porcine teschovirus-1 (PTV-1) 2A autoproteolytic cleavage site (ATNFSLLKQAGDVEENPGP), and the entire ORF of the nuclear export protein (NEP). mCherry and Venus ORFs were cloned using AgeI and NheI restriction sites, into the pDZ or pHW2000 plasmids to generate the pDZ_H1N1_NS-mCherry and pHW_H5N1_NS-Venus for virus rescues. Plasmid constructs were confirmed by complete DNA sequencing (Plasmidsaurus, South San Francisco, USA). Recombinant pH1N1 or HPh-TX H5N1 containing wild-type (WT) NS1 or modified NS1-mCherry or NS1-Venus, respectively, were rescued as previously described [22]. Viruses were aliquoted and stored at −80°C until use. Viral titers were calculated using the standard plaque assay in MDCK cells.

### Human airway organoid (HAO) infection

Commercial human-derived tracheal/bronchial epithelial cells were cultured at an air–liquid interface (ALI) (MatTek, Ashland, USA). These multilayered HAO, composed of ciliated, goblet, basal, and club cells, maintained in provided medium that was changed every 3 days, were cultured into inserts in 6-well tissue plates. The fully differentiated HAO model closely mimics the epithelial architecture of the respiratory tract, exhibiting mucociliary activity and a polarized epithelium representative of the upper respiratory system. The epithelial barrier integrity was monitored by examining the leakage of basal media to the apical part of the insert. There was no leakage observed before or during virus infection, reflecting the self-renewal capacity of the HAO tissue. Before infection, the apical surfaces of HAO were washed twice with PBS to remove accumulated mucus. HAO cultures were infected apically with influenza virus diluted in infection media at a multiplicity of infection (MOI) of 0.01 for short infection (3 days) to assess early host responses and at an MOI of 0.001 for long infection (10 days) to allow multiple rounds of viral replication and to evaluate sustained tissue responses over time. The viral inoculum was incubated on the apical surface for 2 h at 37°C, 5% CO_2_. Following this infection period, the viral inoculum was removed, and the apical surface was washed three times with PBS. Cultures were maintained at 37°C, 5% CO_2_, and viral replication was monitored over time under fluorescence microscopy (EVOS M 5000, Invitrogen, Waltham, USA). At designated post-infection times, apical washes were collected by incubating the apical surface with 200 µL of warm PBS for 30 min at 37°C, followed by collection and storage at −80°C for later viral titration. Basolateral media were also collected for cytokine and chemokine analyses.

### Virus quantification by plaque assay

MDCK cells were seeded in 6-well plates (10⁶ cells/well) and incubated overnight at 37°C in a 5% CO₂ humidified incubator. Confluent monolayers were infected with 10-fold serial viral dilutions for 1 h at 37°C. After viral absorption, cell monolayers were overlaid with agar-containing infection medium and incubated at 37°C, 5% CO₂. After 3 days, cells were fixed overnight with 10% neutral buffered formalin. To visualize viral plaques, wells were stained with 1% crystal violet for 5 min at room temperature (RT) and rinsed with tap water.

### Virus replication kinetics

The secreted mucus from HAO tissues was washed out, and the tissue was infected with rHPh-TX H5N1 or rHPb-TX H5N1 in triplicate at MOI of 0.01 for 2 h. After viral infection, the accumulated mucus was collected from the apical part of the insert, and the presence of infectious virus at 24, 48, and 72 hours post-infection (hpi) was determined by plaque assay in MDCK cells. Viral RNA was extracted from HAO tissues using Trizol and quantified by qRT-PCR. A549 cell monolayers were seeded in 6-well plates (10⁶ cells/well, triplicate) and incubated for 24 h at 37°C in a humidified 5% CO₂ incubator. Cells were then infected with the indicated viruses at an MOI of 0.01 and incubated for 1 h to allow viral adsorption. After adsorption, the virus inoculum was removed, and cells were washed three times with PBS to eliminate non-adsorbed viral particles. Fresh infection medium (3 mL) was added to each well, and plates were incubated at 37°C in a humidified 5% CO₂ incubator. Cell culture supernatants (200 µL per sample) were collected at 24-, 48-, and 72-hpi and replaced with an equal volume of fresh infection medium. Viral titres were calculated using the standard plaque assay in MDCK cells.

### Immunostaining

HAO tissue was washed with warm PBS and fixed in 10% formalin. Following tissue permeabilizing and blocking to reduce non-specific binding, the tissues were incubated with a primary antibody specific to α-SMA conjugated with Alexa Fluor™ 488 overnight (Invitrogen, Waltham, USA, Cat #: 53-9760-82). Residual α-SMA antibody was washed with PBS, and the tissue was incubated for 15 min with a deep red cell mask (Invitrogen, Waltham, USA, Cat #: H32721) for cell shape visualization using fluorescence microscopy for imaging.

### RNA extraction, cDNA synthesis, and qPCR analysis

Total RNA was extracted from the HAO and A549 cells using TRIzol reagent (Invitrogen, Waltham, USA, Cat #: 15596026) following the manufacturer’s instructions. Genomic DNA was removed using the DNase kit (Invitrogen, Waltham, USA, Cat. #:18068015), and RNA quantity and quality were assessed using a nanodrop. According to the manufacturer’s protocol, the cDNA was synthesized using 1 µg of total RNA using an oligo(dT) primer and reverse transcriptase kit (Invitrogen, Waltham, USA, Cat. #: 4368814). Quantitative real-time PCR (qPCR) was performed using SYBR Green Master Mix (Applied Biosystems, Carlsbad, USA, Cat. #: 43-091-55) on an ABI 7500 system. The thermal cycling conditions included 45 cycles of 95°C for 10 s, 60°C for 10 s, and 72°C for 10 s. Relative gene expression was analyzed using the 2^−^ΔΔCt method, with GAPDH as an internal reference gene. Primers used for amplification of genes are shown in Table 1.

**Table 1.** Primers used in this study.

Gene	Primers	Sequence (5’ 3’)
IL6 (NM_000600)	Forward Reverse	AGACAGCCACTCACCTCTTCAG TTCTGCCAGTGCCTCTTTGCTG
IL8 (NM_000584)	Forward Reverse	GAGAGTGATTGAGAGTGGACCAC CACAACCCTCTGCACCCAGTTT
IL1β (NM_000576)	Forward Reverse	CCACAGACCTTCCAGGAGAATG GTGCAGTTCAGTGATCGTACAGG
TNF-α (NM_000594)	Forward Reverse	CTCTTCTGCCTGCTGCACTTTG ATGGGCTACAGGCTTGTCACTC
MMP2 (NM_004530)	Forward Reverse	AGCGAGTGGATGCCGCCTTTAA CATTCCAGGCATCTGCGATGAG
MMP9 (NM_004994)	Forward Reverse	GCCACTACTGTGCCTTTGAGTC CCCTCAGAGAATCGCCAGTACT
IFNβ1(NM_002176)	Forward Reverse	CTTGGATTCCTACAAAGAAGCAGC TCCTCCTTCTGGAACTGCTGCA
ISG15 (NM_005101)	Forward Reverse	CTCTGAGCATCCTGGTGAGGAA AAGGTCAGCCAGAACAGGTCGT
IRF3 (NM_001571)	Forward Reverse	TCTGCCCTCAACCGCAAAGAAG TACTGCCTCCACCATTGGTGTC
NFKB1 (NM_003998)	Forward Reverse	GCAGCACTACTTCTTGACCACC TCTGCTCCTGAGCATTGACGTC
TGFβ1 (NM_000660)	Forward Reverse	TACCTGAACCCGTGTTGCTCTC GTTGCTGAGGTATCGCCAGGAA
COL1A1 (NM_000088)	Forward Reverse	GATTCCCTGGACCTAAAGGTGC AGCCTCTCCATCTTTGCCAGCA
COL3A1 (NM_000090)	Forward Reverse	TGGTCTGCAAGGAATGCCTGGA TCTTTCCCTGGGACACCATCAG
FN (NM_212482)	Forward Reverse	ACAACACCGAGGTGACTGAGAC GGACACAACGATGCTTCCTGAG
IAV M gene*	M30F2/08 M264R3/08	ATGAGYCTTYTAACCGAGGTCGAAACG TGGACAAANCGTCTACGCTGCAG
GAPDH (NM_002046)	Forward Reverse	GTCTCCTCTGACTTCAACAGCG ACCACCCTGTTGCTGTAGCCAA

*IAV universal mixture primers have been validated for all IAV subtypes from human samples.

“Y” refers to C or T; “N” refers to an A, G, C, or T.

### Multiplex immunoassay

The multiplex approach enables efficient use of limited volumes of HAO mucus samples while increasing throughput and reducing assay variability compared to running individual ELISAs. Levels of cytokines (IL-6, IL-1β, TNF-α) and chemokines (IP-10/CXCL-10, RANTES/CCL5) were measured using a custom human ProcartaPlex immunoassay panel (Thermo Fisher Scientific, Waltham, USA, Cat. #: PPX-12-D5619750), following the manufacturer’s protocol. Mucus samples and basal media of HAO were collected at 48 hpi with rHPh-TX H5N1. Samples were centrifuged at 10,000 × *g* for 5 min to remove debris and diluted 1:2 in Universal Assay Buffer before analysis. The assay was conducted under BSL-3 conditions, and samples were decontaminated by overnight incubation in 1% formaldehyde before readout on a Luminex 100/200 System (xPONENT v4.3.309.1). Instrument settings included a gate range of 7,500–25,000, a 50 µL sample volume, 50 events per bead, a 60 s sample timeout, and standard PMT settings. Data were analyzed using xPONENT v4.3.309.1.

### Statistical analysis

Data was analyzed using GraphPad Prism 9.5.1 software. The normality of data distribution was verified using the Shapiro–Wilk test, and homogeneity of variances was verified with Levene’s test. Viral titres, cytokines, chemokines, and fibrosis markers levels were normally distributed, and compared using *t*-test, one-way, or two-way ANOVA with post-hoc corrections as appropriate. *P*-values <0.05 were considered statistically significant.

## Results

### rHPh-TX H5N1 replicated efficiency in HAO

**Figure 1.**
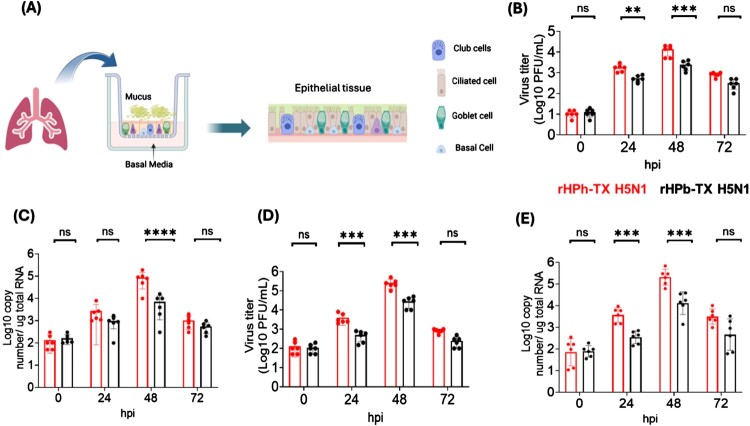
Replication kinetics of rHPh-TX H5N1 and rHPb-TX H5N1 in HAO. (A) A diagram shows HAO at an air-liquid interface (ALI) culture system. The HAO, composed of ciliated, goblet, basal, and club cells, was grown on inserts in 6-well plates. (B) HAO were infected in triplicate at MOI 0.01 with rHPh-TX H5N1 or rHPb-TX H5N1 for 2 h after washing out the secreted mucus. After infection, the accumulated mucus was collected from the apical part of the insert, and the presence of infectious virus at the indicated hours post-infection (hpi) was determined by plaque assay in MDCK cells. (C) HAO-infected tissues were collected for viral RNA extraction and quantified by qRT-PCR. (D) A549 cells were grown in 6-well plates (10⁶ cells/well, triplicate) and infected (MOI 0.01) with rHPh-TX H5N1 or rHPb-TX H5N1 for 1 h. Virus titres in cell culture supernatants were measured by plaque assay in MDCK cells. (E) qRT-PCR was used to measure viral RNA in cell lysates. The rHPh-TX H5N1 exhibited higher replication efficiency than rHPb-TX H5N1 in HAO and A549 cells. Data was analyzed using one-way ANOVA followed by Tukey’s HSD post-test. The significant differences are indicated (** = *p* < 0.01, *** = *p* < 0.001; **** = *p* < 0.0001; non-significant = ns).

Human airway epithelial tissue is the primary site of IAV infection and replication. The air–liquid interface model of HAO (Fig.1A) has been considered a platform to assess viral tropism and infectivity in humans for pandemic risk assessment of zoonotic IAVs [23]. We infected HAO with rHPh-TX H5N1 or rHPb-TX H5N1at MOI 0.01 to examine virus shedding in the secreted mucus and replication efficiency in human epithelial tissues. The viruses exhibited higher replication efficiency in HAO at 48 hpi measured by the levels of infectious particles in the secreted mucus (Figure 1B) and viral RNA levels in infected tissues (Figure 1C). However, rHPh-TX H5N1 exhibited higher replication efficiency than rHPb-TX H5N1, especially at 48 hpi. The reduction in virus titer at 72 hpi could be due to the cytopathic effect caused by virus replication in the ciliated and goblet cells. No infectious virus was detected in the HAO basal media during infection. For comparison, we infected A549 cells with both viruses at MOI 0.01. Similarly, the infectious virus titres in the culture supernatants and viral RNA levels in cell lysates from infected A549 cells were higher at 48 hpi with higher replication efficiency of rHPh-TX H5N1 compared to rHPb-TX H5N1 (Figures 1D and 1E, respectively).

### rHPh-TX H5N1 induces high IFNand pro-inflammatory responses in HAO

**Figure 2.**
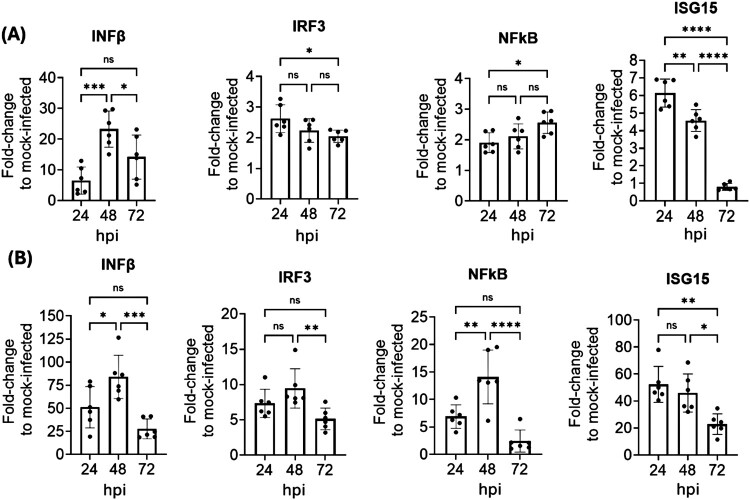
IFN responses to rHPh-TX H5N1 infection in HAO and A549 cells. (A) HAO were mock-infected or infected in triplicate with rHPh-TX H5N1 (MOI 0.01) for 2 h, and the tissues were collected at 24, 48, and 72 hpi for RNA extraction by Trizol. Relative gene expression to mock-infected cells after normalization with the endogenous gene (GAPDH) was determined by qRT-PCR. (B) A549 cells were infected with rHPh-TX H5N1 (MOI 0.01) for 1 h. Mock and virus-infected A549 cells were collected at 24, 48, and 72 hpi for RNA extraction by Trizol, and gene expression levels were determined by qRT-PCR. The rHPh-TX H5N1 induces a robust IF response in HAO and A549 cells, indicating a strong activation of the host's innate immune system. Data was analyzed using One-way ANOVA followed by Tukey’s HSD post-test. The significant differences are indicated (* = *p* < 0.05, ** = *p* < 0.01, *** = *p* < 0.001; **** = *p* < 0.0001; non-significant = ns).

We next examined the expression levels of key factors of the IFN pathway following rHPh-TX H5N1 infection of HAO. Control and infected tissue samples of HAO and A549 cell lysates were collected for RNA extraction and qRT-PCR. Human A549 cells are a well-characterized model for studying innate immune responses to respiratory viruses, including influenza viruses. Their consistent baseline expression and predictable responsiveness to IFN pathways provide a useful reference point for interpreting virus-host interactions. Expression levels of key factors of the antiviral signalling pathways were significantly upregulated upon infection with rHPh-TX H5N1 (Figure 2A). For comparison, we assessed expression levels of the same factors in A549 cells after infection with rHPh-TX H5N1 (Figure 2B). Generally, the IFN response of HAO to rHPh-TX H5N1 infection was lower than in A549 cells, as the HAO cell types might have varied responses representing more accurately the actual response in human airway tissues compared to the synchronized response observed in A549 cells. Indeed, the highest IFNbeta (IFN-β) levels in rHPh-TX H5N1-infected HAO were observed at 48 hpi, ∼20-fold higher than mock-infected HAO (Figure 2A). We observed ∼75-fold induction in infected A549 compared to mock-infected cells (Figure 2B). IFN regulatory factor 3 (IRF3) and nuclear factor-kappa B (NF-κB) showed ∼2-3-fold higher expression levels in rHPh-TX H5N1-infected HAO *vs.* mock-infected when compared to ∼10-15-fold observed in A549 cells (Figures 2A and 2B). IRF3 and NF-κB are critical transcription factors for the induction of type I IFN and pro-inflammatory cytokines. Interestingly, NF-κB significantly increased at 72 hpi in infected HAO; however, it dramatically decreased in A549 cells (Figures 2A and 2B). The upregulation of IRF3 and NF-κB led to higher interferon-stimulated gene 15 (ISG15) expression at 24 hpi in infected HAO (∼6-fold), while in A549 cells it was ∼50-fold (Figures 2A and 2B). Altogether, these results demonstrate that rHPh-TX H5N1 induces a robust IFN response in HAO and A549 cells, indicating a strong activation of the host's innate immune system.

**Figure 3.**
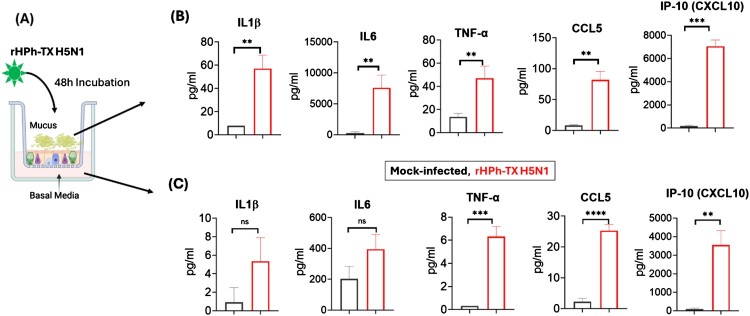
Pro-inflammatory cytokine and chemokine levels in HAO infected with rHPh-TX H5N1. (A) HAO inserts in triplicate were infected with rHPh-TX H5N1 (MOI 0.01) for 48 h. (B) Secreted mucus in the apical part of the insert was collected at 48 hpi by adding 200 µL warm PBS and incubating for 15 min at 37°C. Mucus samples and (C) basal medium were collected at 48 hpi, spun down, and used for the multiple cytokine assay. Data was analyzed using the *t*-test comparison. The significant differences are indicated (** = *p* < 0.01, *** = *p* < 0.001; **** = *p* < 0.0001; non-significant = ns).

To examine the inflammatory status of infected HAO, we measured pro-inflammatory markers in secreted mucus and the basal media using a multiplex cytokine assay (Figure 3A). Results showed elevated levels of pro-inflammatory cytokines such as TNF-α, IL-6, IL-1β, and chemokines CCL5 (RANTES), and IP-10 (CXCL10) after infection with rHPh-TX H5N1. Higher levels of these markers were found in the HAO mucus compared to the basal media, which correlated with the viral titres observed (Figures 3B and C). The excessive production of these mediators suggests that rHPh-TX H5N1 may trigger potent antiviral signalling pathways; however, it may also contribute to immunopathology.

### rHPh-TX H5N1 and pH1N1 infections induce fibrogenesis in HAO

**Figure 4.**
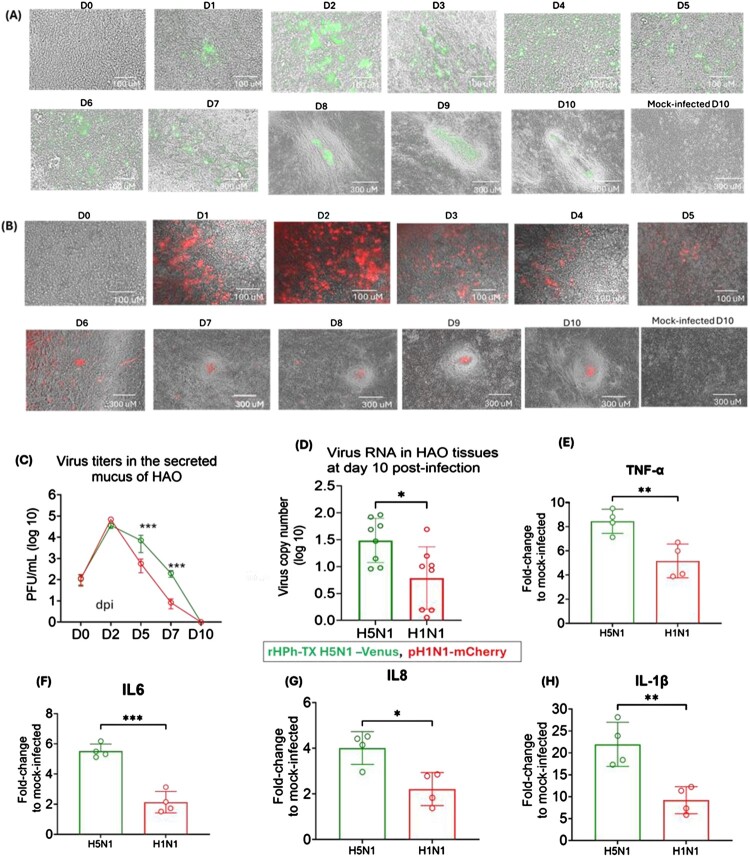
Prolonged infection of HAO with rHPh-TX H5N1-Venus and pH1N1-mCherry induces fibroblast-like cell foci surrounding infected areas and inflammation. (A, B) HAO inserts were infected in triplicate (MOI of 0.001) with (A) rHPh-TX H5N1-Venus or (B) pH1N1-mCherry for 2 h and monitored daily under fluorescence microscopy for 10 days. (C) Virus titres in HAO-secreted mucus were determined by plaque assay. (D) On 10-DPI, virus RNA levels in HAO-infected tissues were determined by qRT-PCR. (E-H) Gene expression levels of pro-inflammatory cytokines in the HAO tissues at 10-DPI. Data was analyzed using two-way ANOVA and the unpaired *t*-test. The significant differences are indicated (* = *p* < 0.05, ** = *p* < 0.01, *** = *p* < 0.001; **** = *p* < 0.0001; non-significant = ns).

HAO derived from bronchial progenitor cells and differentiated to pseudostratified tissue, closely mimics the morphology and function of human airways under near-physiological conditions [24]. Using this model, we next investigated the effects of rHPh-TX H5N1 infection on fibrogenesis activation compared to pH1N1 infection. It has been reported that pH1N1 infections can rapidly progress to ARDS and contribute to pulmonary fibrosis [25]. Pulmonary fibrosis in pH1N1-infected patients is related to the increased activity of fibroblasts in the post-inflammatory repair pathways [26]. To monitor prolonged virus infection in HAO, we generated recombinant pandemic A/California/04/09 H1N1 expressing mCherry (pH1N1-mCherry) and recombinant A/Texas/37/2024 H5N1 expressing Venus (rHPh-TX H5N1-Venus) fused to the C-terminus of NS1 from their modified NS segments. These reporter viruses replicated efficiently in MDCK cells, achieving viral titres of ∼10^7^ PFU/ml (rHPh-TX H5N1-Venus) and ∼10^6^ PFU/ml for pH1N1-mCherry (data not shown). The reporter rHPh-TX H5N1-Venus and pH1N1-mCherry were used to monitor infection dynamics in HAO over 10 days (Figures 4A and B). Both recombinant viruses express different fluorescent proteins to eliminate any possible interaction of the reporter genes with the formation of fibroblast-like cell foci. We infected HAO with rHPh-TX H5N1-Venus and pH1N1-mCherry at an MOI of 0.001. Peak viral replication of these viruses was observed on day 2 post-infection (2-DPI), as determined by fluorescent expression, followed by a gradual decline over the subsequent days (Figures 4A and B). By 7-DPI, consolidation areas enriched with elongated fibroblast-like cells were evident, surrounding the infected regions. The fibroblast-like cells progressively accumulated, isolating infected areas from the surrounding tissue, potentially reflecting localized host responses to infection. These structures were prominent at the latest times post-infection (e.g. 7-DPI to 10-DPI) (Figures 4A and B). Viral titres in secreted mucus were measured by plaque assay using MDCK cells (Figure 4C). The highest viral titer for both viruses was at 2-DPI, declining to undetectable levels at 7- to 10-DPI. Interestingly, viral RNA was detected at 10-DPI despite the absence of infectious virus in mucus (Figure 4D). Consistently, we observed increased expression of pro-inflammatory cytokine responses in the HAO tissue harvested on 10-DPI, reflecting inflammatory status (Figures 4E-H). TNF-α, IL-6, IL-8, and IL-1β showed higher levels in prolonged infection of HAO with rHPh-TX H5N1-Venus *vs.* pH1N1-mCherry (Figures 4E-H). These data indicate that after 10-DPI, viral RNA can still be detected in HAO despite the lack of infectious virus in secreted mucus, with consistent increased expression of pro-inflammatory cytokines.

### rHPh-TX H5N1 and pH1N1 infections upregulated fibrogenesis markers in HAO

**Figure 5.**
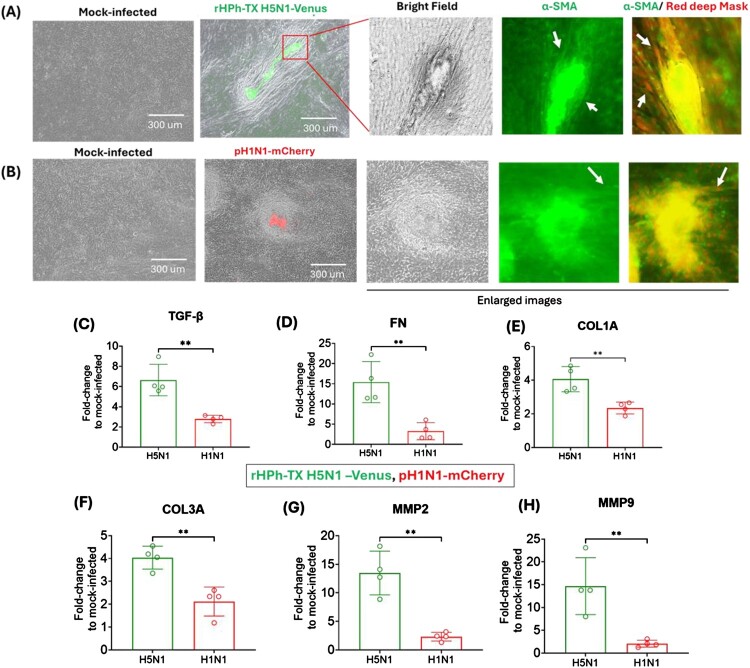
High expression of α-SMA after prolonged infection of HAO with HPh-TX H5N1-Venus and pH1N1-mCherry. (A, B) HAO were separately infected in triplicate (MOI of 0.001) with (A) HPh-TX H5N1-Venus and (B) pH1N1-mCherry for 2 h. At 10-DPI, HAO tissue was washed 2X with PBS, fixed with 10% formaldehyde, permeabilized with 0.5% Triton-100, and incubated with Alexa Fluor 488-labelled α-SMA antibody overnight. The tissue was incubated with a red deep cell mask for 15 min to visualize the cell shape. (C-E) Gene expression of TGF-β and extracellular matrix proteins FN, COL1A, and COL3A. (F,G) Gene expression tissue remodelling MMP2 and MMP9. Data were analyzed using an unpaired *t*-test, ** = *p* < 0.01.

Our data showed alteration in the HAO epithelial tissue's structure by forming fibroblast-like cells surrounding the virus-infected area during rHPh-TX H5N1 and pH1N1 infection. To investigate fibrogenesis activation, we next evaluate whether rHPh-TX H5N1 and pH1N1 infections induce fibroblast differentiation into myofibroblasts by α-SMA. Fluorescent staining revealed extensive FITC signal in fibroblast-like cell foci, with α-SMA filaments localized with spindle-like cell shapes, confirming the myofibroblast phenotype surrounding virus-infected cells (Figures 5A and 5B). These activated fibroblasts are characterized by spindle morphology with intracytoplasmic stress fibres, a contractile phenotype, expression of various mesenchymal immunocytochemical markers like α-SMA, and collagen production [27]. The spindle morphology of fibroblasts and the high level of α-SMA expression suggest fibroblast-to-myofibroblast differentiation as part of tissue remodelling. The epithelial–mesenchymal transition (EMT) has recently received consideration as a process by which epithelial cells lose cell–cell attachment, polarity, and epithelial-specific markers, undergo cytoskeletal remodelling, and gain a mesenchymal phenotype [28,29]. TGF-β induces epithelial–mesenchymal alterations and myofibroblast transformation that contribute to lung fibrosis [20]. rHPh-TX H5N1-Venus or pH1N1-mCherry caused upregulation of TGF-β gene expression at 10-DPI, compared to mock-infected HAO (Figure 5C). However, rHPh-TX H5N1-Venus induced ∼7-fold higher expression of TGF-β, while pH1N1-mCherry only induced ∼2.5-fold higher expression of TGF-β (Figure 5C). Besides the role of TGF-β as a key profibrotic cytokine-promoting fibroblast proliferation, TGF-β is one of the most important stimulators of ECM production [30]. As such, we evaluated gene expression of ECM coding genes, including FN, COL1A, and COL3A, in HAO at 10-DPI with rHPh-TX H5N1-Venus or pH1N1-mCherry. We observed a ∼15-fold increase in FN expression caused by rHPh-TX H5N1-Venus infection and a ∼2.5-fold increase in pH1N1-mCherry-infected HAO (Figure 5D). Similarly, COL1A and COL3A increased ∼4-fold by rHPh-TX H5N1-Venus infection and ∼2-fold during pH1N1-mCherry infection (Figures 5E and F). These increase in the ECM gene expression was combined with upregulation of tissue remodelling matrix metalloproteinase MMP2 (Figure 5G) and MMP9 (Figure 5H) enzymes that were upregulated ∼15-fold after rHPh-TX H5N1-Venus infection *vs.* ∼2-fold upregulation in pH1N1-mCherry-infected HAO. These results suggest that rHPh-TX H5N1 infection induces a more pronounced EMT and ECM remodelling than pH1N1, as evidenced by the significantly higher upregulation of TGF-β and ECM-associated genes. The robust increase in FN, COL1A, and COL3A expression, along with the marked upregulation of MMP2 and MMP9, highlights the potential for H5N1 to drive a profibrotic response in infected epithelial tissues. Given the well-established role of TGF-β in promoting fibrosis [20], these findings suggest that H5N1 infection may contribute to long-term lung remodelling and fibrotic pathology through sustained EMT and ECM deposition. Understanding these mechanisms could provide insight into the differential pathogenicity of IAV strains and inform targeted therapeutic strategies to mitigate fibrosis-associated complications.

### ROCK1 activity is required for fibroblast-like cell foci formation caused by HPh-TX H5N1 infection in HAO

**Figure 6.**
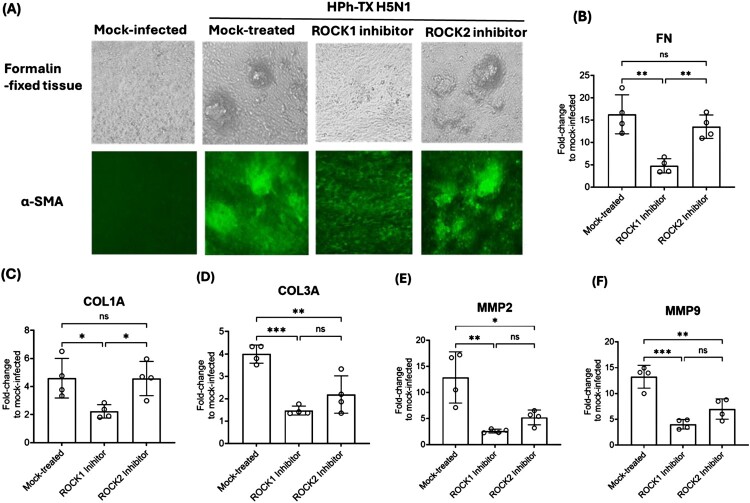
Inhibition of ROCK1 impaired fibroblast-like cell foci formation after infection of HAO with HPh-TX H5N1. (A) HAO tissue was infected in triplicate (MOI 0.001) with HPh-TX H5N1 and treated with a non-toxic dose (5 μM) of ROCK1 (GSK269962) or ROCK2 (KD025, Belumosudil) inhibitors added to the basal media from 5-DPI to 10-DPI. HAO tissue was fixed and stained with α-SMA (green) at 10-DPI. (B-D) Gene expression of extracellular matrix proteins FN, COL1A, and COL3A. (E, F) Gene expression of tissue remodelling MMP2 and MMP9. Data was analyzed using One-way ANOVA followed by Tukey’s HSD post-test. The significant differences are indicated (* = *p* < 0.05, ** = *p* < 0.01, *** = *p* < 0.001; non-significant = ns).

Our data suggest that H5N1 infection caused higher HAO remodelling and upregulation of the fibrotic cytokines and ECM deposition compared to pH1N1. These findings align with the long-term consequences of H5N1 pathogenicity, such as pneumonia, lung fibrosis, and respiratory failure [31,32]. Unlike pH1N1, which typically causes self-limiting respiratory illness in immunocompetent individuals, H5N1 infection is associated with severe lung injury, prolonged inflammation, and a higher likelihood of developing post-viral fibrosis [16,26]. As such, we sought a therapeutic approach to tackle the H5N1 pathogenesis. The ROCK1 and ROCK2 signalling pathways play a central role in regulating cytoskeletal dynamics, EMT, and ECM remodelling that are critically involved in fibrogenesis [20]. We investigated the role of ROCK isoforms in HAO fibrosis after infection with a HPh-TX H5N1 natural isolate. Using natural virus isolate better reflects the genetic makeup of the circulating strain, making findings more translatable to clinical settings. To this end, we evaluated the effects of selective ROCK1 (GSK269962A) and ROCK2 (Belumosudil/KD025) inhibitors on the fibroblast-like cell foci formation after infecting HAO (MOI 0.001) with HPh-TX H5N1 for 10 days. Infected HAO were treated with a non-toxic dose of ROCK1 and ROCK2 inhibitors (5 µM) added to the basal media for 5 days, starting from 5-DPI to 10-DPI, the period of fibroblast-like cell foci formation (Figure 4A). On 10-DPI, the tissue was collected, fixed, and immunostained with a FITC-conjugated antibody against α-SMA (Figure 6A). ROCK1 inhibitor-treated HAO showed reduced α-SMA expression, with no detectable fibroblast-like foci formation. In contrast, ROCK2 inhibitor-treated HAO exhibited higher α-SMA expression and retained fibroblast-like foci (Figure 6A). We also measured the gene expression of the ECM protein, which showed a significant reduction of FN, COL1A, and COL3A in ROCK1-treated HAO compared to mock-treated or ROCK2-treated tissues (Figures 6B–D). We also observed a significant decrease in ECM-modulating enzymes MMP2 (Figure 6E) and MMP9 (Figure 6F) in ROCK1-treated HAO *vs.* mock-treated or ROCK2-treated tissues. These results suggest that inhibition of ROCK1, but not ROCK2, effectively suppresses fibroblast-like foci formation and ECM remodelling in HPh-TX H5N1-infected HAO. The reduction in α-SMA expression and the downregulation of FN, COL1A, and COL3A in ROCK1 inhibitor-treated HAO indicate that ROCK1 plays a pivotal role in driving EMT and fibrosis. Furthermore, ROCK1 inhibition significantly reduced MMP2 and MMP9 expression, suggesting reduced ECM degradation and remodelling. In contrast, ROCK2 inhibition failed to prevent fibroblast-like foci formation and was associated with sustained ECM protein expression, highlighting the distinct roles of ROCK isoforms in fibrosis.

## Discussion

The unpredictable evolution of IAVs makes future pandemics inevitable, highlighting the urgent need for preparedness to mitigate health, social, and economic impacts. H5N1 cases were first described in Hong Kong in 1997 when birds died from an outbreak of the disease, propagating to humans later in the same year [33,34]. Since the resurgence of H5N1 in 2002–2003, global efforts have been intensified to prevent its spread. The emergence of HPh/b-TX H5N1 represents a significant zoonotic threat, with its origin linked to infected dairy cows with highly pathogenic H5N1 from the clade 2.3.4.4b [35]. This outbreak underscores the risk of cross-species transmission as the virus has spread within poultry, cattle, and humans, posing a significant public health threat [36].

Infections with H5N1 cause severe pneumonia that can progress to lung fibrosis and respiratory failure [31,32]. While current FDA-approved antivirals reduce viral loads [37], these antivirals are often ineffective in preventing lung injury and fibrosis, underscoring the need for novel therapeutic strategies targeting host responses. To better understand viral replication and pathogenesis, we employed HAO, a physiologically relevant model that recapitulates key aspects of the human lung environment.

We infected HAO with either rHPh-TX H5N1 or rHPb-TX H5N1 at MOI of 0.01 to assess viral replication efficiency. Our findings demonstrate that rHPh-TX H5N1 replicates more efficiently in HAO, serving as a surrogate for human airway epithelial tissues, compared to rHPb-TX H5N1. The elevated levels of infectious rHPh-TX H5N1 particles detected in secreted mucus indicate a greater degree of viral adaptation to the human airway epithelium. This efficient replication and shedding into mucus may facilitate aerosolization and increase the potential for person-to-person transmission. Additionally, the robust replication of rHPh-TX H5N1 in HAO was associated with a markedly strong induction of the IFN response, reflecting heightened host recognition and immune activation. Typically, H5N1 infection activates the RIG-I/MAVS signalling pathway, leading to IRF3 and NF-κB activation, which drives IFN-β production and ISG15 expression [38–40]. Our data show that rHPh-TX H5N1 induces IRF3 and NF-κB expression, which increase IFN-β and ISG15 levels. While ISG15 contributes to antiviral defense, its dual role in immune regulation can either enhance or suppress IRF3/NF-κB signalling, potentially influencing disease severity. In severe cases, H5N1 evades IFN pathways, leading to excessive NF-κB-driven inflammation (cytokine storm) and impaired IRF3-mediated responses, exacerbating lung damage [41–43]. Cytokine profiling of infected HAO revealed that rHPh-TX H5N1 induced elevated IL-6, IL-1β, TNF, CCL5, and IP-10 (CXCL10) levels. While these cytokines aid in viral clearance, prolonged inflammation may contribute to tissue injury and fibrosis (e.g. cytokine storm-induced lung injury).

Given the robust inflammatory response induced by rHPh-TX H5N1, we investigated its role in airway fibrogenesis during prolonged infection with rHPh-TX H5N1 compared to seasonal pH1N1. Our results indicate that prolonged infection of HAO with rHPh-TX H5N1 and pH1N1 induced fibroblast-like cells surrounding the infected area, associated with high cytokine response and α-SMA expression, indicating fibroblast-to-myofibroblast differentiation. It has been reported that H1N1 infections can rapidly progress to ARDS and contribute to pulmonary fibrosis [25]. Furthermore, severe IAV infections are associated with higher levels of TGF-β, indicating a relationship between disease severity and IAV-induced pulmonary fibrosis [44]. This agrees with our findings showing that the rHPh-TX H5N1 infection induces higher TGF-β and ECM-associated gene expression than pH1N1. As such, the increased fibroblast activity in post-inflammatory repair pathways, with a pivotal role played by TGF-β and ECM, seems to be linked to IAV-induced pulmonary fibrosis.

We hypothesize that the EMT process plays a prominent role in fibrogenesis. During the EMT process, epithelial cells lose cell–cell attachment, polarity, and epithelial-specific markers, undergo cytoskeletal remodelling, and gain a mesenchymal phenotype [45]. Our results indicate that rHPh-TX H5N1 infection induces more pronounced EMT and ECM remodelling than pH1N1, as evidenced by the significantly higher upregulation of TGF-β and ECM-associated genes. We also observed the spindle shape of fibroblasts with intensive expression of α-SMA. In this regard, activated fibroblasts are described as spindle or stellate morphology cells with intracytoplasmic stress fibres, a contractile phenotype, expression of various mesenchymal markers such as α-SMA, and collagen production [27].

We also observed that at 10-DPI, pro-inflammatory (TNF, IL-6, IL-8, IL-1β) and pro-fibrotic (TGF-β) mediators were upregulated, along with ECM components (FN, COL1A, COL3A, MMP2, and MMP9). Importantly, the upregulation of FN expression allows microbes to adhere to epithelial surfaces and contributes to the virulence of secondary bacterial infections [46,47]. Collectively, these factors contribute to a feedback loop of persistent lung damage and fibrosis [26], emphasizing the need for targeted interventions to disrupt these pathways. Our data showed that the ROCK pathway affects fibroblast foci formation and ECM deposition. The ROCK1 and ROCK2 pathways are key regulators of fibrosis with distinct roles in tissue remodeling [20]. Our results indicate that the inhibition of ROCK1 activity significantly reduced fibroblast foci formation and ECM deposition, whereas ROCK2 inhibition had a lesser effect. This suggests a dominant role for ROCK1 in airway fibrosis during H5N1 infection. Previous studies described the capacity of antifibrotic agents such as pirfenidone and nintedanib, FDA-approved to treat idiopathic pulmonary fibrosis (IPF), to reduce fibrosis through inhibiting key cytokines, including TGF-β and vascular endothelial growth factor (VEGF) [48,49]. Thus, unravelling the mechanisms of the ROCK signalling pathway in IAV-induced fibrogenesis may be essential for developing effective strategies to prevent and treat viral-induced pulmonary fibrosis, ultimately mitigating its long-term impact on respiratory health.

To our knowledge, this is the first study to characterize the profibrotic response triggered by the highly pathogenic HPh-TX H5N1 strain in human airway organoids. Our organoid model recapitulates the structural and functional complexity of the human airway epithelium, including multicellular composition and differentiation markers that are absent in traditional immortalized cell lines [50]. Unlike animal models, this system provides a controlled, human-specific platform to study early epithelial responses to viral infection [51]. The novelty of our approach is further underscored by using a recent zoonotic H5N1 isolate (HPh-TX H5N1), associated with the 2024 human and bovine outbreaks in the U.S., which has not previously been evaluated in this context. While our study provides insights into H5N1 pathogenesis and triggering fibrogenesis, it also has limitations. Though physiologically relevant, the HAO model does not fully recapitulate *in vivo* immune responses or systemic factors that promote disease progression. HAO lacks immune cell components, limiting its ability to model immune cell recruitment and systemic cytokine amplification. While our findings demonstrate strong epithelial-intrinsic responses to rHPh-TX H5N1, they may underestimate the full extent of cytokine storm observed *in vivo*. Future studies should evaluate the therapeutic potential of ROCK1 inhibitors in preclinical models of IAV-induced lung injury and fibrosis. Investigating the molecular links between IFN signalling, inflammation, and fibrotic remodelling will uncover additional therapeutic targets. Although our findings support the regulatory role of ROCK1, further investigation of downstream effectors, such as SMAD4 and p-MLC [52,53], is required to clarify the mechanisms underlying ROCK1-mediated fibrotic and inflammatory responses. Future studies will include examining the fibrotic potential of other highly pathogenic IAV with zoonotic potential, such as H7N9. Additionally, we will assess the effects of currently approved antiviral therapies, such as neuraminidase inhibitors (e.g. Oseltamivir) and endonuclease inhibitors (e.g. Baloxavir), in combination with ROCK1 inhibitors, which could provide additive or synergistic benefits by simultaneously controlling virus-induced lung injury and mitigating fibrotic responses.

In conclusion, our findings highlight the efficient replication and innate immunity immune activation of H5N1 in HAO. H5N1 induces a robust inflammatory and fibrotic response, driven by NF-κB and TGF-β signalling, contributing to airway remodelling and fibrosis. ROCK1 inhibition is a promising therapeutic strategy that warrants further investigation in appropriate *in vivo* models. These insights emphasize the importance of developing host-targeted therapies to prevent severe lung complications associated with influenza infections.

## Authors contributions

Conceptualization: H.R. and L.M-S.; Methodology: H.R., A.M., M.B., C.Y., R.S.B., and A.A-G.; Data collection and interpretation: H.R. and L.M-S.; Funding acquisition and resources: H.R. and L.M-S.; Writing, review, and editing: H.R., A.N.J., J.B.T., and L.M-S.; all authors have read and agreed to the published version of the manuscript.
